# *HLA-G* 3’UTR polymorphism diplotypes and soluble HLA-G plasma levels impact cervical cancer susceptibility and prognosis

**DOI:** 10.3389/fimmu.2022.1076040

**Published:** 2022-12-21

**Authors:** Jun Gan, Xing-Hong Di, Zi-Yi Yan, Yang-Fan Gao, Hui-Hui Xu

**Affiliations:** ^1^ Medical Research Center, Taizhou Hospital of Zhejiang Province, Wenzhou Medical University, Linhai, Zhejiang, China; ^2^ School of Clinical Medicine, Wenzhou Medical University, Wenzhou, Zhejiang, China; ^3^ Key Laboratory of Minimally Invasive Techniques & Rapid Rehabilitation of Digestive System Tumour of Zhejiang Province, Linhai, China

**Keywords:** HLA-G, 3’UTR, diplotypes, sHLA-G, cervical cancer

## Abstract

**Background:**

Human leukocyte antigen G (HLA-G) is an immune checkpoint molecule with relevance in several cancers. The aim of this study was to evaluate the potential role of soluble HLA-G (sHLA-G), its genetic polymorphisms and its haplotype structure in the susceptibility and prognosis of primary cervical cancer in a Chinese Han population.

**Methods:**

We investigated sHLA-G plasma levels and 3’ untranslated region (3’UTR) polymorphisms through ELISA and direct DNA sequencing, respectively, in cervical cancer patients (120 cases) and healthy control women (96 cases). The data were analyzed for associations using PowerMarker, Haploview, and GraphPad Prism.

**Results:**

In this study, 8 polymorphic sites, 16 haplotypes and 23 diplotypes in the *HLA-G* 3’UTR were identified in our study population. We observed that each pair of 8 polymorphic sites exhibited linkage disequilibrium. The heterozygote CT genotype at position +3422 (rs17875408) was more common in cervical cancer patients than in healthy women (OR=5.285, *P*<0.05). Haplotypes UTR-1, UTR-3, and UTR-7 accounted for more than 85% of both groups, but no significant difference was found. The frequency of the UTR-1/UTR-3 diplotype in patients was significantly higher than that in controls (*P*<0.05). In addition, we further observed that *HLA-G* 3’UTR polymorphisms may influence the sHLA-G plasma level in patients’ peripheral blood, especially *14 bp Ins/Del* (rs371194629) and +*3142 C/G* (rs1063320). A receiver operating characteristic (ROC) curve analysis showed that the sHLA-G level had good diagnostic performance in differentiating patients with cervical cancer from healthy women (AUC>0.7). Among patients, mean sHLA-G levels increased with increasing FIGO stages but were not related to the overall survival time.

**Conclusions:**

The results of the present study enhance our understanding of how *HLA-G* 3’UTR polymorphisms can influence the peripheral sHLA-G plasma level and play a key role in cervical carcinogenesis. This study further confirmed that sHLA-G may represent a novel plasma biomarker for the prognosis and potential therapeutic target of cervical cancer.

## Background

Cervical cancer is a common malignant tumor in females that originates in the cervix. Persistent infection with human papillomavirus (HPV) leads to almost all cervical cancers and their precursors (1). Human leukocyte antigen G (HLA-G) is an immunosuppressive molecule involved in the escape mechanisms of virus-infected and malignant cells. Our previous studies showed that *HLA-G* polymorphism was a susceptibility factor for active HPV infection, especially high-risk HPV types (2, 3). We speculate that HPV infection may escape host immunosurveillance by inducing HLA-G expression in cervical epithelial cells (4). An increasing number of studies have confirmed that HLA-G is an immune checkpoint molecule with immunosuppressive and carcinogenic activities (5, 6). HLA-G expression is strongly related to higher tumor grade and worse prognosis for patients with cervical cancer (7–10).

HLA-G is a nonclassical HLA class I molecule, and its coding gene is located on chromosome 6p21.3, which has limited polymorphisms. To date, only 110 alleles and 36 proteins have been identified (http://hla.alleles.org/nomenclature/stats.html, September 2022). *HLA-G* mRNA is alternatively spliced, resulting in at least four membrane-bound (HLA-G1, -G2, -G3, -G4) and three soluble (HLA-G5, -G6, -G7) protein isoforms (11, 12). Membrane-bound HLA-G1 isoform can be cleaved by metalloproteinases, producing soluble shedding HLA-G1 (sHLA-G1). Soluble HLA-G (sHLA-G) in peripheral blood mainly includes sHLA-G1 and HLA-G5 isoforms, which maybe expressed and released by cancer cells in the tissue microenvironment (10). It has been found that polymorphic sites may affect the expression of HLA-G molecules by changing the affinity of gene-targeted sequences or modifying the stability of *HLA-G* mRNA or the binding sites of microRNAs (13, 14). Most of the relevant studies have focused on the role of 3’ untranslated region (3’UTR) polymorphisms of the *HLA-G* gene, particularly *14 bp insertion/deletion (Ins/Del)* (5’-ATTTGTTCATGCCT-3’, rs371194629) and +*3142 C/G* (rs1063320) (2, 3, 15). Our previous studies showed that *14 bp Ins* or *+3142 G* alleles increased the risk of Chinese women acquiring cervical HPV (2, 3). The *14 bp Ins* allele produces an additional splice, removing 92 bp, which may affect *HLA-G* mRNA stability and cause a decrease in sHLA-G levels in plasma (16, 17). The *+3142 G* allele favors binding miR-148a, miR-148b, and miR-152, causing increased degradation of *HLA-G* mRNA (18). It was previously reported that the expression of HLA-G in cervical intraepithelial neoplasia (CIN) with HPV16/18 infection was significantly higher than that in CIN without HPV infection, and the sHLA-G plasma level was significantly higher in CIN and SCC patients (8, 9). The above data indicate that the genetic heterogeneity of the *HLA-G* gene may lead to heterogeneous HLA-G expression in different populations. Therefore, the *HLA-G* 3’UTR polymorphisms of women’s genetic backgrounds were related to susceptibility to HPV infection, and we speculated that *HLA-G* 3’UTR polymorphisms may affect the development of cervical carcinogenesis by regulating sHLA-G level. However, the distribution of HLA-G alleles, genotypes, and haplotypes in cervical cancer patients and their possible roles in the expression of HLA-G levels remain unclear. The aim of this study was to investigate the association between *HLA-G* 3’UTR polymorphisms and sHLA-G plasma levels in Chinese Han cervical cancer patients. In addition, the association between the sHLA-G level and the diagnosis and prognosis of cervical cancer was also investigated.

## Materials and methods

### Study population

Since 2008, cervical cancer patients have been recruited at Taizhou Hospital, which is affiliated with Wenzhou Medical University, China. In total, 120 patients who were initially diagnosed with cervical cancer were enrolled. The exclusion criteria were radiotherapy, chemotherapy, or other medical interventions before blood collection. Staging of cervical cancer was performed according to the International Federation of Gynaecology and Obstetrics (FIGO) criteria, and patients were classified into Stages I, II, III, and IV. Follow-up was performed on 21 July 2022. In addition, 96 unrelated healthy women who were negative for HPV were also enrolled in this study. All included subjects were of Chinese Han descent.

This study was approved by the Medical Ethics Review Committee of Taizhou Hospital (approval #K20220226), and written informed consent from all study participants was obtained before enrolment.

### *HLA-G* gene 3’UTR analysis

The NCBI reference sequence (RefSeq: NC_000006.12) was used as the standard for primer design and sequence alignment in this study. Genomic DNA was extracted using the commercially available DNA Extraction Kit (#GK0122, GENEray, China). The *HLA-G* gene 3’UTR was amplified by PCR (forward primer: 5’-TGAGCATGTGATGGGCTGTT-3’ and reverse primer: 5’-GGGAAGAGGTGTAGGGGTCT-3’). The PCR amplification product had a length of 704 bp or 718 bp, encompassing nucleotides +2846 to +3563 with 23 single nucleotide polymorphism (SNP) sites (rs371194629, rs567747015, rs1707, rs1710, rs17179101, rs146339774, rs17179108, rs569057854, rs180827037, rs554784083, rs138249160, rs1063320, rs554076817, rs187320344, rs9380142, rs1610696, rs1233331, rs541542414, rs556033566, rs561554956, rs530204611, rs17875408, rs190871790). In addition, we performed another PCR with primers 14F and 14R to validate the *14 bp Ins/Del* polymorphism in each sample as previously described (2).

The PCR mixture included 25 μL 2×PrimeSTAR Max Premix (#R040A, TaKaRa, Japan), 20 μL nuclease-free water, 2 μL forward primer, 2 μL reverse primer, and 1 μL template DNA in a final volume of 50 μL. The PCR conditions were 95°C for 5 min; 35 repeated cycles of 94°C for 45 s, 62°C for 45 s and 72°C for 1 min; and a final extension step at 72°C for 7 min. Subsequently, the PCR amplification products were purified and sequenced using the reverse primer on an ABI 3730XL instrument at BGI Company. HLA-G polymorphic sites were analyzed by interpreting the chromatogram peaks using BioEdit.

### sHLA-G measurement

Plasma was separated and stored at -80°C until sHLA-G level determination. The concentration of sHLA-G (U/mL) in the peripheral plasma of cervical cancer patients and healthy women was quantified by ELISA kit (Cat# RD194070100R, sHLA-G kit; BioVendor) using our previously described method (10). In this ELISA kit, combinations monoclonal antibody (mAb) MEM-G/09 (capture) + anti-β2m (detection) were chosen for the simultaneous determination of shed HLA-G1 and soluble HLA-G5.

### Statistical analysis

*HLA-G* allele, genotype, haplotype and diplotype frequencies were compared between patients with cervical cancer and healthy women using the chi-squared (χ^2^) test. Hardy−Weinberg equilibrium for *HLA-G* genotypic data was estimated using the χ^2^ test with 1 df (SPSS 23.0). Linkage disequilibrium (LD) between each pair of polymorphic sites was evaluated using PowerMarker 3.25 and Haploview 4.2. The sHLA-G concentration was analyzed using the Mann−Whitney U test and Spearman rank correlation test (GraphPad Prism 5.0). Survival probabilities were calculated using Kaplan−Meier analysis. A receiver operating characteristic (ROC) curve was used to evaluate the diagnostic performance of sHLA-G concentration to discriminate between patients and healthy women. A two-sided *P* value less than 0.05 was considered statistically significant.

## Results

### Characteristics of the study population

A total of 120 patients (58.1 ± 13.5 years; range, 33~91 years) with an initial diagnosis of cervical cancer were included; 108 (90.0%) patients also had squamous cell carcinoma (SCC), 11 (9.2%) patients had adenocarcinoma (ADC) and 1 (0.8%) patient had adenosquamous carcinoma (ASC). The follow-up period was 14 years (range, 2~174 months) or until death. The clinical characteristics of the study population are summarized in **Table 1**.

**Table 1:** Study Population Characteristics.

Variables	Cervical Cancer patients (n=120)	Healthy women (n=96)
**Age (years)**		
Mean ± SD	58.1 ± 13.5	43.2 ± 8.5
Range	33 ~ 91	24 ~ 69
**Histological types**		
Squamous cell carcinoma (SCC)	108 (90.0%)	/
Adenocarcinoma (ADC)	11 (9.2%)	/
Adenosquamous carcinoma (ASC)	1 (0.8%)	/
**FIGO Stage**		
I	27 (22.5%)	/
II	59 (49.2%)	/
III	33 (27.5%)	/
IV	1 (0.8%)	/
**Nodal status**		
Negative	42 (35.0%)	/
Positive	16 (13.3%)	/
Unknown	62 (51.7%)	/
**Follow-up**		
alive	86 (71.7%)	96 (100%)
death	34 (28.3%)	0

Of the twenty-three SNP sites, only eight were recognized in the study population as 3’UTR polymorphisms of the *HLA-G* gene. We subsequently analyzed eight polymorphic sites, including *14 bp Ins/Del* (rs371194629), *+3010 C/G* (rs1710), *+3027 A/C* (rs17179101), *+3035 C/T* (rs17179108), *+3142 C/G* (rs1063320), *+3187 A/G* (rs9380142), *+3196 C/G* (rs1610696), and *+3422 C/T* (rs17875408). All HLA-G genotypic frequencies fit Hardy−Weinberg equilibrium (data not shown).

### Analysis of 3’UTR polymorphisms in the *HLA-G* gene

The allelic and genotypic frequencies observed in patients and healthy women are summarized in **Table 2**. Of the 8 SNP sites, a significant difference in genotypic frequencies was observed only in the heterozygote CT genotype at position +3422 (rs17875408), which was more frequent in cervical cancer patients than in healthy women (OR=5.285, *P*=0.039).

**Table 2:** Comparisons of the *HLA-G* 3’UTR allelic and genotypic frequencies between patients and controls.

SNP	Allele/Genotype	Cervical Cancer Patients		Heathy women		*P* value
		Count	Frequency	Count	Frequency	
rs371194629	14bp Del	164	0.6833	130	0.6771	
	14bp In	76	0.3167	62	0.3229	0.890
	14bp Del/Del	56	0.4667	46	0.4792	
	14bp Int/Del	52	0.4333	38	0.3958	
	14bp Int/Int	12	0.1000	12	0.1250	0.780
rs1710	+3010 C	138	0.5750	111	0.5781	
	+3010 G	102	0.4250	81	0.4219	0.948
	+3010 CC	39	0.3250	37	0.3854	
	+3010 CG	60	0.5000	37	0.3854	
	+3010 GG	21	0.1750	22	0.2292	0.235
rs17179101	+3027 A	54	0.2250	44	0.2292	
	+3027 C	186	0.7750	148	0.7708	0.918
	+3027 AA	5	0.0417	6	0.0625	
	+3027 AC	44	0.3667	32	0.3333	
	+3027 CC	71	0.5917	58	0.6042	0.727
rs17179108	+3035 C	183	0.7625	147	0.7656	
	+3035 T	57	0.2375	45	0.2344	0.939
	+3035 CC	71	0.5917	57	0.5938	
	+3035 CT	41	0.3417	33	0.3438	
	+3035 TT	8	0.0667	6	0.0625	0.992
rs1063320	+3142 C	99	0.4125	80	0.4167	
	+3142 G	141	0.5875	112	0.5833	0.930
	+3142 CC	18	0.1500	21	0.2188	
	+3142 CG^a^	63	0.5250	38	0.3958	
	+3142 GG	39	0.3250	37	0.3854	0.146
rs9380142	+3187 A	141	0.5875	112	0.5833	
	+3187 G	99	0.4125	80	0.4167	0.930
	+3187 AA	39	0.3250	37	0.3854	
	+3187 AG^b^	63	0.5250	38	0.3958	
	+3187 GG	18	0.1500	21	0.2188	0.146
rs1610696	+3196 C	220	0.9167	174	0.9063	
	+3196 G	20	0.0833	18	0.0938	0.704
	+3196 CC	100	0.8333	78	0.8125	
	+3196 CG	20	0.1667	18	0.1875	
	+3196 GG	0	0	0	0	0.689
rs17875408	+3422 C	172	0.7167	140	0.7292	
	+3422 T	68	0.2833	52	0.2708	0.773
	+3422 CC	59	0.4917	55	0.5729	
	+3422 CT^c^	54	0.4500	30	0.3125	
	+3422 TT	7	0.0583	11	0.1146	0.071

All HLA-G genotypic frequencies fit the Hardy-Weinberg equilibrium expectations (*P*>0.05, data not shown).

^a^χ^2^=3.574, *P*=0.059.

^b^χ^2^=3.574, *P*=0.059.

^c^χ^2^=4.243, *P*=0.039.

**Figure 1:**
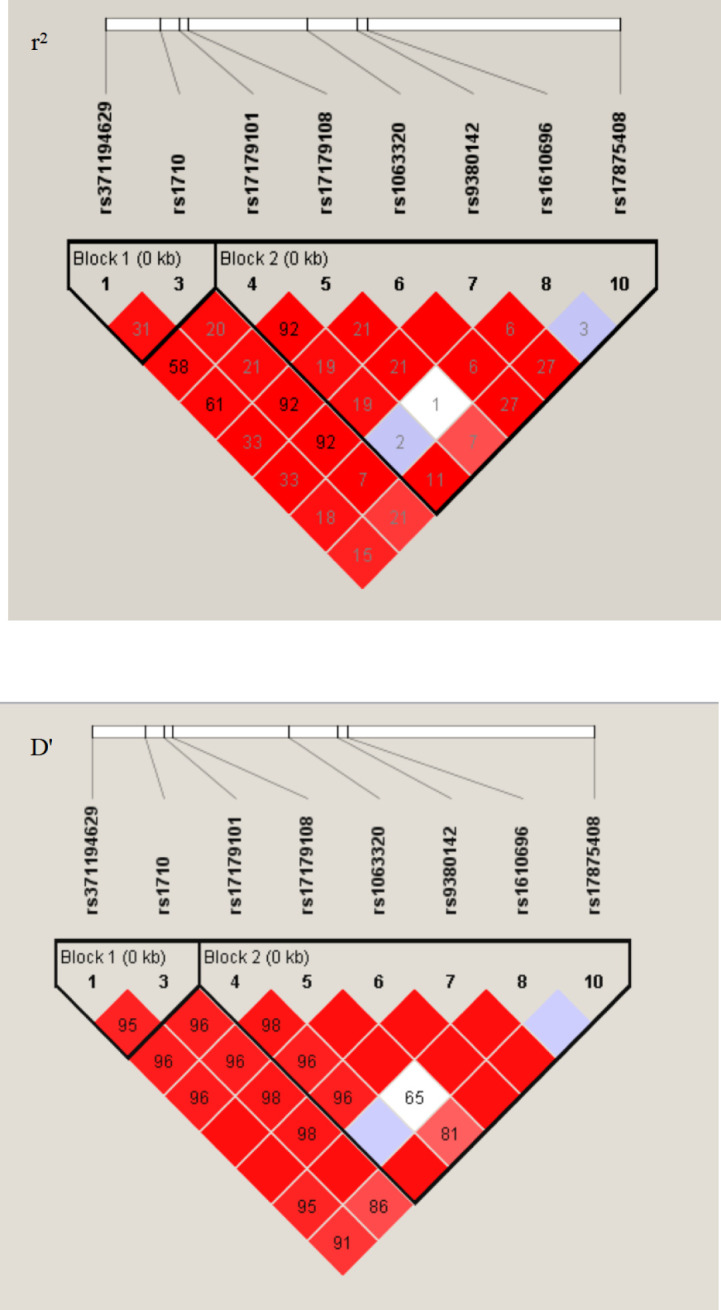
LD patterns between eight pairs polymorphic sites at the 3’UTR region of *HLA-G* gene. The image was generated in the Haploview 4.2. D’ and r^2^, pairwise correlation between polymorphic sites. Areas in red indicated strong LD (r^2^>0.8), shades of pink indicated moderate LD, and white indicates low LD. The r^2^ values (×100) for the marker pairs are listed in the corresponding boxes.

To investigate whether haplotypes are more predictive of cancer susceptibility than single SNP sites, we performed a haplotype linkage analysis on alleles. Among the study population, 16 different haplotypes were generated using PowerMarker software. These haplotypes were named in this study and were consistent with those previously published, according to the worldwide distributions addressing the *HLA-G* 3’UTR polymorphic sites (19). The results showed that the haplotype frequencies of UTR-1, UTR-3, and UTR-7 accounted for more than 85% of both groups. However, no significant differences were observed between patients and healthy women (*P*>0.05) (**Table 3**). As depicted in **Figure 1**, all eight polymorphic sites exhibited linkage disequilibrium (LD). A high LD was observed, especially among the *14 bp Ins, +3142 G* and *+3187 A* alleles, with rare exceptions. In addition, a nearly perfect LD was observed between *+3027 A/C* and *+3035 C/T* alleles, *+3010 C/G* and *+3142 C/G* alleles, and *+3010 C/G* and *+3187 A/G* alleles (D’=0.98, r^2 =^ 0.92).

**Table 3:** *HLA-G* 3’UTR haplotype frequencies among patients and controls.

Haplotypes^#^		Cervical Cancer patients		Healthy women		*P* value
		Count	Frequency	Count	Frequency	
UTR-1	Del-G-C-C-C-G-C-C	98	0.4083	79	0.4115	0.948
UTR-3	Del-C-C-C-G-A-C-T	62	0.2583	46	0.2395	0.655
UTR-7	Ins-C-A-T-G-A-C-C	52	0.2166	42	0.2187	0.958
UTR-2	Ins-C-C-C-G-A-G-C	19	0.0792	17	0.0885	0.726
UTR-21	Del-G-C-C-G-A-C-T	2	0.0084	2	0.0104	0.822
Others*	/	7	0.0292	6	0.0313	0.912

*P* values were calculated using χ^2^ test.

^#^HLA-G 3'UTR haplotyprs were named according to the worldwide distributions (19).

*group of all haplotypes with number was one.

Haplotype consensus sequences are represented by: +2960 14 bp In/Del l (rs371194629), +3010 C/G (rs1710), +3027 A/C (rs17179101), +3035 C/T (rs17179108), +3142 C/G (rs1063320), +3187 A/G (rs9380142), +3196 C/G (rs1610696), and +3422 C/T (rs17875408).

We further analyzed 23 different diplotypes in the study population. The most common diplotypes in cervical cancer patients were UTR-1/UTR-3 (24.2%), UTR-1/UTR-7 (19.2%) and UTR-1/UTR-1 (14.2%), while the most common diplotypes in healthy women were UTR-1/UTR-1 (20.8%), UTR-1/UTR-7 (15.6%) and UTR-1/UTR-3 (11.5%) (**Table 4**). The frequency of the UTR-1/UTR-3 diplotype in patients was significantly higher than that in healthy women (*P*<0.05). These findings further confirmed that HLA-G genetic polymorphism was related to the risk of cervical cancer in the Chinese Han population in the Taizhou area, especially in women with diplotype UTR-1/UTR-3, which may favor susceptibility to cervical cancer.

**Table 4:** *HLA-G* 3’UTR diplotype frequencies among patients and controls.

Diplotypes	Cervical Cancer patients		Heathy women		*P* value
	Count	Freqency	Count	Freqency	
UTR1/UTR3	29	0.2417	11	0.1146	0.017
UTR1/UTR7	23	0.1917	15	0.1563	0.497
UTR1/UTR1	17	0.1417	20	0.2083	0.196
UTR3/UTR7	11	0.0917	10	0.1042	0.758
UTR2/UTR3	8	0.0667	4	0.0417	0.425
UTR3/UTR3	7	0.0583	10	0.1042	0.214

### sHLA-G plasma levels in cervical cancer patients and healthy women

**Figure 2:**
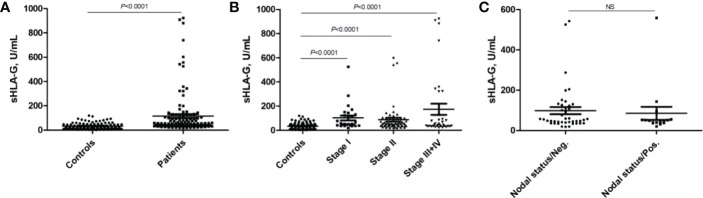
Comparison of sHLA-G plasma levels of cervical cancer patients and healthy women. **(A)** sHLA-G is significantly (*P*<0.0001) elevated in patients, **(B)** sHLA-G levels increase with ascending FIGO stage in patients without reaching significance. **(C)** sHLA-G levels based on nodal status. Bars indicate mean ± SEM. Statistic was performed by Mann-Whitney test. NS, not significant.

**Figure 3:**
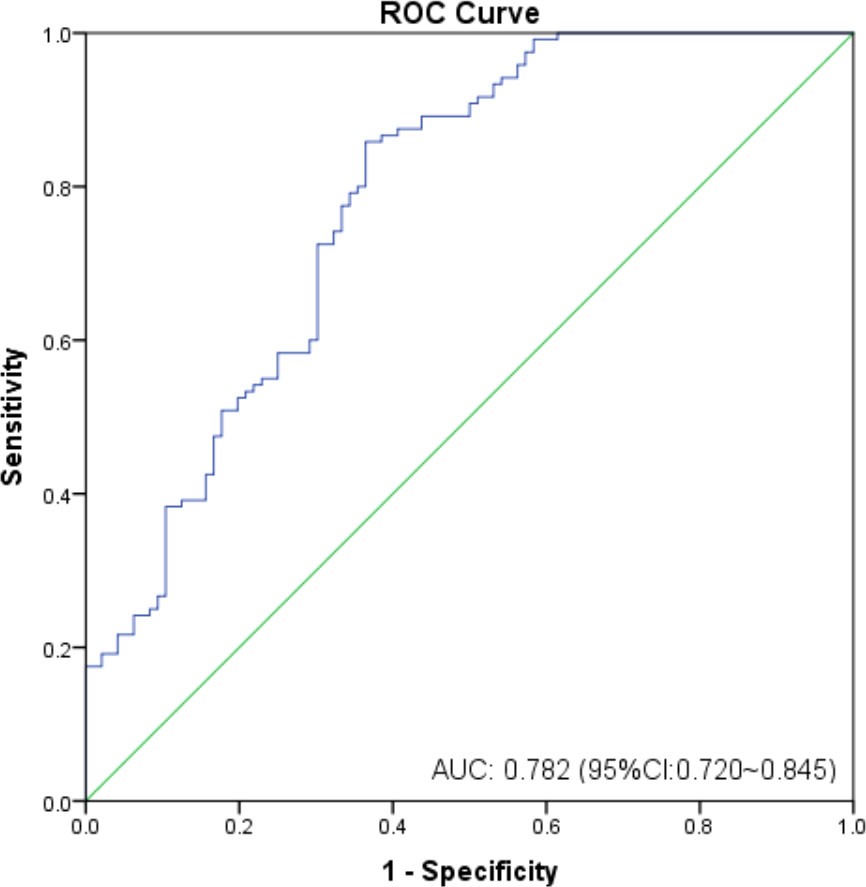
ROC analysis of sHLA-G between cervical cancer patients and healthy women controls.

As shown in **Figure 2**, the mean sHLA-G level in patients’ peripheral blood was almost threefold higher than that in healthy women (*P*<0.0001, 115.80 ± 177.6 *vs.* 32.99 ± 26.74 U/mL), regardless of *HLA-G* polymorphisms. We further analyzed whether sHLA-G levels could differentiate cervical cancer patients from healthy women by ROC analysis (**Figure 3**). The results showed that sHLA-G had good diagnostic performance in differentiating patients with cervical cancer (AUC=0.782). The ROC curve showed that at a threshold value of 33.75 U/mL, the diagnostic sensitivity and specificity of sHLA-G for cervical cancer were 86% and 64%, respectively. Among patients, the sHLA-G plasma level increased with increasing FIGO stage, but the difference was not significant (*P*>0.05). Compared with healthy women, the sHLA-G plasma level in the patients with stage I were significantly higher (*P*<0.001). Therefore, sHLA-G can be also considered a supplementary biomarker for the early diagnosis of cervical cancer.

Patients were grouped according to the sHLA-G cut-off value, and no significant difference was found in the overall survival time between patients with low sHLA-G levels (< 33.75 U/mL) and those with high sHLA-G levels (> 33.75 U/mL). Survival analysis showed that there was no correlation between the sHLA-G plasma level and the overall survival time of patients.

### Analysis of sHLA-G plasma levels based on HLA-G genetic polymorphisms

**Figure 4:**
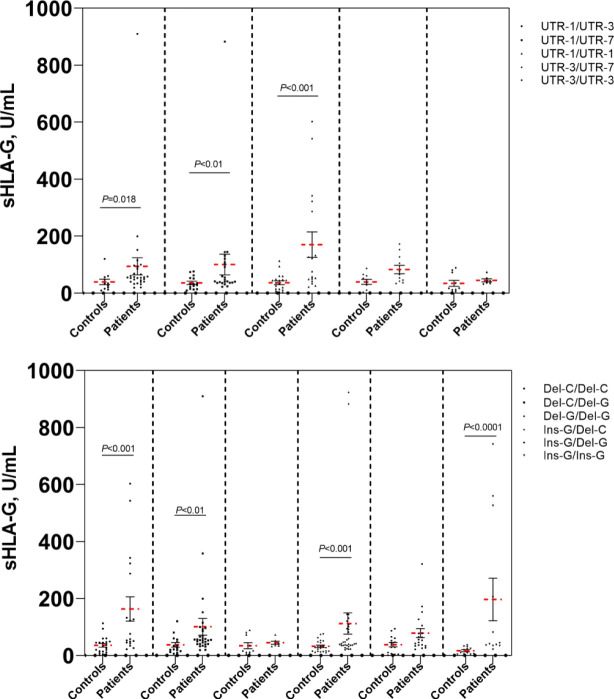
Comparison of sHLA-G levels of cervical cancer patients and healthy controls. Bars indicate mean ± SEM.

To further investigate whether the plasma sHLA-G levels were affected by the patients’ genetic background of *HLA-G* polymorphic sites, we analyzed sHLA-G plasma levels in patients’ peripheral blood according to *HLA-G* polymorphism. As depicted in **Figure 4**, we compared the sHLA-G levels between *HLA-G* 3’UTR diplotypes and observed difference. Patients with the *Del-3142C/Del-3142C* diplotype had higher sHLA-G production than patients with other diplotypes. We observed that the sHLA-G level increased in patients with the *Del-3142C/Del-3142C* diplotype (76.63 ± 61.71 U/mL) compared to patients with the *Ins-3142G/Ins-3142G* diplotype (60.34 ± 48.58 U/mL) without significance. These results confirmed that HLA-G polymorphisms may influence sHLA-G levels in patients’ peripheral blood. None of the *HLA-G* 3’UTR diplotypes were related to the overall survival time of patients.

## Discussion

HLA-G is an inhibitory checkpoint molecule, which is highly expressed in gynecological cancers, such as cervical cancer (3, 4, 7–10), ovarian cancer (20), breast cancer (21, 22), and endometrial cancer (23). It’s known that virus-infected cells and malignant cells escape the host’s immune surveillance by inducing the expression of HLA-G (4–6, 10). The *HLA-G* 3’UTR contains several SNP sites related to *HLA-G* mRNA stability that impact HLA-G expression (13–19, 24, 25). It has been reported that HLA-G/sHLA-G is highly expressed in the tissues or peripheral blood of cervical cancer patients, contributing to the immune escape of cancer cells (4, 7–10). Here, we focused on evaluating the possible association of *HLA-G* 3’UTR polymorphisms with sHLA-G plasma levels in the Chinese Han population and its value in clinical prognostic evaluation and survival outcome prediction in patients with cervical cancer. In the present study, we observed that (i) the frequency of the heterozygote CT genotype at position +3422 (rs17875408) was significantly higher in cervical cancer patients than in healthy women (*P*<0.05), suggesting that the *+3422 C/T* genotype may be a highly significant risk factor for cervical cancer; (ii) the frequency of the UTR-1/UTR-3 diplotype in patients was significantly higher than that in healthy women (*P*<0.05), which may play a key role in cervical carcinogenesis; (iii) *HLA-G* 3’UTR polymorphic sites may influence the sHLA-G plasma level in patients’ peripheral blood, especially *14 bp Ins/Del* (rs371194629) and +*3142 C/G* (rs1063320); and (iii) the sHLA-G level had good diagnostic performance in differentiating patients with cervical cancer from healthy women, suggesting that sHLA-G can be a supplementary biomarker of cervical cancer.

In this study, we identified eight alleles, sixteen haplotypes and twenty-three diplotypes in the *HLA-G* gene 3’UTR in our Taizhou population. Each of these different haplotypes was in LD with specific *HLA-G* 3’UTR alleles. A high LD among these alleles was detected, especially among *14 bp Ins*, *+3142 G* and *+3187 A*. These alleles have previously been reported to have lower levels of *HLA-G* mRNA, indicating that their effects are not independent, thereby leading to decreased HLA-G expression (16–18, 24, 25). Our results were consistent with this conclusion; we observed that the *14 bp Ins* allele was related to low sHLA-G production and that the *14 bp Del* allele was related to high sHLA-G production (**Figure 4**). Previous studies have shown that elevation of plasma sHLA-G levels is common in patients with various kinds of tumors (10, 20, 24, 26, 27). Notably, sHLA-G plasma levels decreased significantly within 30 days after radical hysterectomy in our previous study (10). This findings confirmed that the source of plasma sHLA-G was mainly expressed and released by cancer cells in the tissue microenvironment. Therefore, we proposed that the increase in sHLA-G was regulated not only by the immune microenvironment of patients but also by their own genetic background. In addition, several studies have debated the regulation of sHLA-G levels by *HLA-G* 3’UTR polymorphisms. These disputes may vary among different populations with different genetic backgrounds (17, 28, 29).

UTR-1 was the most common haplotype (41.2%), followed by UTR-3 (24.0%), UTR-7 (21.9%), UTR-2 (8.9%) and UTR-21 (1.0%) in patients from the Taizhou area, Southeast China. The order of haplotype frequencies in our Taizhou population was almost consistent with the frequencies previously reported in populations from South China, which were UTR-1 (43.0%), UTR-3 (26.5%), UTR-7 (22.0%), UTR-2 (6.5%) and UTR-4 (2.0%) (19). In the Taizhou population, UTR-1 and UTR-3 with the *14 bp Del* allele were the most common haplotypes, which were theoretically considered to exhibit greater sHLA-G production than those with the *14 bp Ins* allele. As shown in **Figure 4**, the mean sHLA-G level in patients with the UTR-1/UTR-1 diplotype was almost twofold higher than that in healthy women with the UTR-1/UTR-1 diplotype. The results suggested that the peripheral sHLA-G level of cancer patients was also regulated by other clinical factors. Our previous study showed that sHLA-G expression may be induced by cytokines in the peripheral blood circulation (10). UTR-2 and UTR-7 with *14 bp Ins, +3142 G* and *+3187 A* alleles may be related to lower sHLA-G levels (17, 19, 30–32). Of note, our results showed that the frequency of the UTR-1/UTR-7 diplotype in patients was significantly higher than that in healthy women (*P*<0.05). HPV-infected women with the UTR-1/UTR-7 diplotype may be more susceptible to cervical cancer.

In addition, this study investigated the performance of the sHLA-G level in the diagnosis of cervical cancer patients and determined that it has a good diagnostic performance (AU-ROC > 0.7). sHLA-G can be considered a supplementary biomarker of cervical cancer. When the patients were grouped according to the sHLA-G cut-off value, no significant difference was found in the overall survival time between patients with low sHLA-G levels (< 33.75 U/mL) and those with high sHLA-G levels (> 33.75 U/mL). Survival analysis showed that there was no correlation between the sHLA-G plasma level and the overall survival time of patients.

The limitations of this study are as follows: (i) lack of identification of HLA-G dimers and monomers in the peripheral plasma of cervical cancer patients; such analysis might help to corroborate the function of different HLA-G polymers; and (ii) lack of identification of shed HLA-G1 and soluble HLA-G5 in sHLA-G; assessing different sHLA-G isoforms might help for development of immunotherapy for cervical cancer.

In conclusion, our study provides evidence that certain *HLA-G* 3’UTR polymorphisms can influence sHLA-G levels in cancer patients’ peripheral blood, especially *14 bp Ins/Del* (rs371194629) and *3142 C/G* (rs1063320). Our previous and current findings enhance our understanding of *HLA-G* polymorphisms, which are not only associated with susceptibility to HPV infection but also involved in the susceptibility to and progression of cervical cancer by affecting sHLA-G levels. Particular *HLA-G* diplotypes are related to the cervical cancer outcome. In addition, the present study further confirmed that sHLA-G may represent a novel plasma biomarker for the prognosis and potential therapeutic target of cervical cancer.

## Data availability statement

The original contributions presented in the study are included in the article/supplementary materials. Further inquiries can be directed to the corresponding author.

## Ethics statement

This study was approved by the Medical Ethics Review Committee of Taizhou Hospital (approval #K20220226), and written informed consent from all study participants was obtained before enrolment.

## Author contributions

All authors listed have made a substantial, direct, and intellectual contribution to the work, and approved it for publication.
